# Supplementary material for: Exploring individual fixel-based white matter abnormalities in epilepsy
Source: Brain Commun. 2023 Dec 22;6(1):fcad352. doi: 10.1093/braincomms/fcad352 (PMC10768884; doi:10.1093/braincomms/fcad352)
Supplement: fcad352_Supplementary_Data [file fcad352_supplementary_data.docx]

**Supplementary Material**


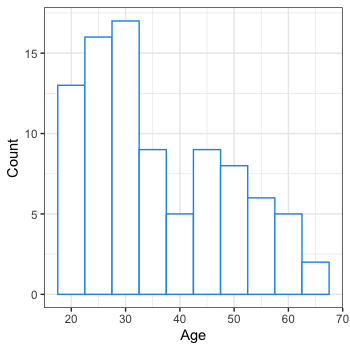


**Supplementary Figure 1: Histogram showing age distribution of healthy control cohort.** Ages shown for healthy control cohort in histogram plot (bin width of 5 years).
